# Integrated Omics Analysis Reveals Alterations in the Intestinal Microbiota and Metabolites of Piglets After Starvation

**DOI:** 10.3389/fmicb.2022.881099

**Published:** 2022-06-15

**Authors:** Yijia Ma, Chang Lu, Bingzhen Ji, Junjun Qin, Chunbo Cai, Yang Yang, Yan Zhao, Guoming Liang, Xiaohong Guo, Guoqing Cao, Bugao Li, Pengfei Gao

**Affiliations:** College of Animal Science, Shanxi Agricultural University, Taigu, China

**Keywords:** starvation stress, microbial diversity, metabolome, ileum, piglet

## Abstract

Obesity is a serious public health problem. Short-term starvation is an effective way to lose weight but can also cause harm to the body. However, a systematic assessment of the relationship between the intestinal microbiota and metabolites after complete fasting is lacking. Pigs are the best animal models for exploring the mechanisms of human nutrition digestion and absorption, metabolism, and disease treatment. In this study, 16S rRNA sequencing and liquid chromatography-mass spectrometry were used to analyze the changes in the intestinal microbiota and metabolite profiles in piglets under starvation stress. The results show that the microbial composition was changed significantly in the starvation groups compared with the control group (*P* < 0.05), suggesting that shifts in the microbial composition were induced by starvation stress. Furthermore, differences in the correlation of the intestinal microbiota and metabolites were observed in the different experimental groups. Starvation may disrupt the homeostasis of the intestinal microbiota and metabolite profile and affect the health of piglets. However, piglets can regulate metabolite production to compensate for the effects of short-term starvation. Our results provide a background to explore the mechanism of diet and short-term hunger for intestinal homeostasis.

## Introduction

Obesity is one of the most serious public health problems in the 21st century (Grace, 2020), and the global prevalence of childhood obesity is increasing annually (Weihrauch-Blüher et al., 2019). In a mouse model induced by a high-fat diet for 16 weeks, it was found that food intake and leptin levels decreased and body fat increased compared with the low-fat diet group, and their energy intake exceeded the basic energy consumption, causing an imbalance in body energy, eventually leading leads to obesity (Choi et al., 2015). Many studies in both rodent models and patients have indicated that obesity is associated with at least 20 different diseases (Weisberg et al., 2003; Reilly and Saltiel, 2017; Wilcock and Haboubi, 2020). Thus, “weight loss” has become a popular solution to this problem. Interventions in obesity management mainly include reducing high-calorie diets and improving physical activity. However, extreme weight loss solutions, including fasting, spontaneous vomiting, very low-calorie diets, and the use of laxatives, have been reported in young adults (Neumark-Sztainer et al., 2011). Mice studies have shown that starvation not only increases the levels of corticosterone, leptin, adrenal cortex hormones, and muscle atrophy but also increases the risk of diseases such as hypoglycemia, hyperkalemia, and hypoacidemia (Lee et al., 2015; Cuevas-Fernández et al., 2019). However, previous studies have shown that dietary restriction can positively affect cardiometabolic function in patients with obesity (Dote-Montero et al., 2022). Foreign researchers have found that short-term fasting can destroy the living environment of cancer cells and reduce the side effects of chemotherapy in patients with breast cancer (Buono and Longo, 2018). Furthermore, fasting has a potential role in reducing oxidative stress damage (Grundler et al., 2020) and inflammation (Brocker et al., 2020), optimizing energy metabolism (Lowe et al., 2020), and delaying aging (Green et al., 2022).

The intestine plays an important role in the digestion and absorption of nutrients and regulation of the endocrine and immune systems (Gasbarrini et al., 2008). Most nutrients are digested and absorbed in the small intestine (Jersild and Clayton, 1971; Quin et al., 1995). The intestinal microbiota contains many bacterial cells, most of which provide essential body functions (Cani and Delzenne, 2007). However, dietary changes can affect the composition and function of the intestinal microbiota. For example, a study showed that a low-fat/high-fiber diet increased the abundance of probiotics (*Lactobacillus* and *Bifidobacterium)* compared with a high-fat/low-fiber diet and stimulated the production of short-chain fatty acids to maintain intestinal homeostasis. On the contrary, a high-fat/low-fiber diet increased the abundance of harmful bacteria *(Escherichia coli* and *Salmonella enterica)*, leading to intestinal digestive disorders in pigs (Heinritz et al., 2016). A previous study indicated that resveratrol could increase the abundance of *Blautia* in mice, alleviate the response to intestinal stress induced by a high-fat diet, and reduce the enrichment of disease-related metabolic pathways (Wang et al., 2020). Interestingly, a very low-calorie diet increased the abundance of *Clostridioides difficile* in the intestine of postmenopausal women, which can affect the body's energy balance by reducing the intestinal absorption of nutrients (von Schwartzenberg et al., 2021).

With the development of high-throughput sequencing technology, using multiple omics analysis is progressing, providing information on the physiology and mechanisms of animal and cell hosts (Fan and Pedersen, 2021). For example, 16s rRNA and metabonomic analysis revealed increased abundance of *Bilophila wadsworthia, Desulfovibrio*, and *Clostridium inocuum*, as well as inhibition of the tricarboxylic acid cycle after fecal transplantation from malnourished twins to mice (Smith et al., 2013). This result provides insight into the occurrence of malnutrition. In a study of non-obese patients with non-alcoholic fatty liver disease, the abundance of members from the families *Ruminococcaceae* and *Veillonellaceae* were related to bile and propionic acids, and it was speculated that they were the main taxa related to the severity of liver fibrosis (Lee et al., 2020). In the intestines of fasting mice, the abundance of Bacteroides increased, whereas acetic and lactic acids decreased, which was associated with the browning of fat (Li et al., 2017). Moreover, the abundance of Firmicutes increased, and Bacteroides and Verrucomicrobia decreased in intermittent fasting mice, which was positively correlated with the production of taurosulfodeoxycholate, which activates *TGR5* to prevent diabetic retinopathy (Eleni et al., 2018).

Mice are important animal models for investigating the molecular basis and pathology of human diseases because of their low feed cost and controllability. However, metabolic and physiological changes in mice differ from those in humans, which reduces the predictive value of clinical research. Compared with mice, the pathology, physiological response, and diet of pigs are more similar to those of humans (Lagrone et al., 2010; Groenen et al., 2012; Perleberg et al., 2018). Therefore, pigs are the best animal models for exploring the mechanisms underlying human nutrient digestion and absorption, metabolism, and disease treatment. Most studies have focused on dietary restrictions (Xu et al., 2018; Castro-Barquero et al., 2020). Only a few have focused on the stress response of humans or animals under complete starvation (Yang et al., 2021). In this study, Large White piglets were selected as research subjects after weaning (28 d). Furthermore, starvation stress models (animals starved for 48 and 72 h, respectively) were established. The effects of starvation on intestinal homeostasis in piglets were investigated based on intestinal microbiota diversity and changes in intestinal metabolites. This study can provide a reference to explore the mechanisms of diet and short-term hunger in intestinal homeostasis.

## Materials and Methods

### Animal Breeding

Animal experiments were approved by the Ethics Committee of the Shanxi Agricultural University (Taigu, China) and performed in accordance with the “Regulations on the Administration of Experimental Animals” (State Council, Beijing, revised in March 2017). The approval number for the Ethics Committee agreement was SXAU-EAW-2018P002005. Large White piglets were raised at the Animal Science and Technology Experimental Station of Shanxi Agricultural University and fed standard diets in accordance with the feeding standards of swine (NY/T 65-2004) issued by the Ministry of Agriculture of the People's Republic of China. Eighteen piglets with similar birth weight (8.54 ± 0.27 kg) at 28 days of weaning were selected for the starvation stress experiment, and the animals were transferred to a clean pig house with a 7-day feeding transition period. After the pre-feeding period, six piglets were randomly selected for euthanasia as the control group (NC). The remaining 12 piglets were randomized, with six individuals starved for 48 or 72 h (ST_48 and ST_72, respectively). All piglets could drink water during the experiments.

### Sample Processing

Anterior venous blood was collected from the piglets, and serum was extracted before euthanasia. Animals were sacrificed at different time points (control and starved groups) according to standard procedures. The ileal contents of the piglets were collected from the control and the experimental groups, which were labeled as ICCT, ICST-48, and ICST-72, respectively. All samples were stored at −80°C until further analysis.

### Determination of Biochemical Indicators in Blood Samples

An enzyme-linked immunosorbent assay kit (Shanghai ELISA Biotechnology Co., Ltd., Shanghai, China) was used to determine serum levels of cortisol and immunoglobulin A (IgA). The above kits were tested according to the following steps. After serum separation, blank well, standard well and sample well were set on the enzyme plate. In addition to blank well, add enzyme standard reagent; Incubate at 37°C for 60 min; Wash solution rest for 30 s and discard, repeat 3–5 times; Chromogenic agent 50 μL, avoid light for 15 min at 37°C; Stop liquid 50 μL, test on machine immediately. Other biochemical indices, including triglycerides (TG), total cholesterol (TC), low-density lipoprotein cholesterol (LDL-C), high-density lipoprotein cholesterol (HDL-C), alkaline phosphatase (AKP), and acid phosphatase (ACP), were measured using an enzyme-labeled instrument (Nanjing Jiancheng Biotechnology Co., Ltd., Nanjing, China). The above kits were tested according to the following steps. Add 2.5 μL distilled water to the blank well of the enzyme plate, 2.5 μL calibrator to the calibration well and 2.5 μL sample to the sample well; 250 μL working solution to all well; After incubation at 37°C for 10 min, the absorbance was measured by the machine.

### DNA Extraction and 16S RRNA Gene Sequencing

DNA was extracted from ileal samples using the QIAamp Fast DNA Stool Mini Kit (Qiagen, Hilden, Germany). The concentration of the extracted DNA was measured using an ultra-micro nucleic acid analyzer (Nano-400; Hangzhou Allsheng Instruments Co., Ltd., Hangzhou, China), wherein the range of OD 260/280 was between 1.80 and 2.00. The V3–V4 region of the microbial 16S rRNA gene was amplified, and index-PCR was performed using the primers 341F (CCTACGGGNGGCWGCAG) and 805R (GACTACHVGGGTATCTAATCC). The PCR products were purified using the Bio-Rad CFX Connect fluorescent quantitative PCR instrument (Bio-Rad Laboratories, Hercules, CA, USA) for quantification. The purified products were mixed in equal amounts, and sequencing adapters were attached to construct a sequencing library. The V3–V4 region of the 16s rRNA was sequenced using the Illumina PE250 Hiseq platform (Illumina, San Diego, CA, USA).

### Metabolic Profiling

#### Sample Preparation

Fifty milligrams of each sample were weighed and transferred to 1.5-mL Eppendorf tubes. The samples were vortexed for 30 s, homogenized at 45 Hz for 4 min, and sonicated for 5 min in an ice-water bath after adding 1 mL of the extracting solvent (2:2:1 acetonitrile:methanol:water, containing internal standard). Homogenization and sonication were repeated thrice, and then the sample was incubated at −20°C for 1 h and centrifuged at 2,000 × *g* at 4°C for 15 min. The resulting supernatants were transferred to liquid chromatography-mass spectrometry (LC-MS) vials and stored at −80°C until ultra-high performance liquid chromatography (UHPLC)-QE orbitrap/MS analysis. Samples for quality control were prepared by mixing equal aliquots of the supernatant from all samples.

#### LC-MS Analysis

LC-MS analyses were performed using a 1,290 UHPLC system (Agilent Technologies, Santa Clara, CA, USA). The mobile phase comprised solvent A (0.1% formic acid +5 mmol/L ammonium acetate) and B (acetonitrile). The elution gradient was established as follows: 0 min, 1% B; 1 min, 1% B; 8 min, 99% B; 10 min, 99% B; 10 min, 1% B; 12 min, 1% B. The flow rate was 0.5 mL/min, and the injection volume was 2 μL. In the LC/MS experiment, a QE mass spectrometer was used to obtain the MS/MS spectra based on the information dependence basis. In this mode, the acquisition software (Xcalibur 4.0.27; Thermo Fisher Scientific, Waltham, MA, USA) continuously evaluates the full scan measured MS data when collecting and triggering MS/MS spectral acquisition according to pre-selected criteria.

#### Data Preprocessing and Annotation

MS raw data files were processed using the R package XCMS (version 4.2.1), including retention time alignment, peak detection, and peak matching. Each sample was normalized. The internal MS/MS database of OSI-SMMS (Dalian Chem Data Solution Information Technology Co., Ltd., Dalian, China) was used for peak annotation after data processing.

#### Bioinformatics Analysis

The 16S rRNA sequence data were optimized and selected to generate an operational taxonomic unit (OTU) cluster and perform a species taxonomy analysis. We selected the Tag sequence with the highest abundance in OTUs and used the naive Bayesian assignment algorithm of the RDP classifier for these representative sequences. The reference database was used to identify each representative sequence (the confidence threshold was set to 0.8–1.0). Alpha and beta diversity indices were calculated using Quantitative Insights into Microbial Ecology software (version 1.8.0) based on OTU counts and visualized using R (version 4.2.1). The screening of differential microbiota based on intestinal microbiota changes was calculated using the Kruskal–Wallis test and visualized using R (version 4.2.1). Principal component analysis (PCA) was conducted using the R package “ggbiploy” for all samples. Correspondence analysis (CCA) was performed based on the relative abundances of microbial species at different taxa levels using the R package “vegan.” Co-occurrence analysis was performed by calculating Spearman's rank correlations between the predominant taxa, and the R package “heatmap” was used to display the associations among taxa. In addition, the potential Kyoto Encyclopedia of Genes and Genomes (KEGG) ortholog functional characteristics of the microbial community were predicted using PICRUSt and visualized using the R package “ggplot2.”

PCA analysis of metabonomic data was conducted similarly to the 16S rRNA sequencing analysis procedure. The Orthogonal projection to latent structures-discriminant analysis (OPLS-DA) model was further validated by cross-validation. For cross-validation, the data was partitioned into seven subsets and used as a validation set. When the prediction power (Q2) value was between 0.4 and 0.9, it was considered to be a valid prediction model. In the OPLS model, the variable projection importance (VIP) score was used to rank the metabolites with the best distinction between groups (the threshold of VIP was set to 1). The *t*-test was also used as a univariate analysis to screen for differential metabolites. Data with *P* < 0.05 and VIP > 1 were considered significantly different. Metabolites were mapped to KEGG metabolic pathways for enrichment analyses and visualized using the R package “ggplot2.” The pathways screened with a threshold of FDR ≤ 0.05 were considered to be significantly different metabolite enrichment pathways. Spearman's correlation analysis was used to correlate bacterial genera and different metabolites, and the R package “heatmap” was used to display the correlation.

### Statistical Analysis

All statistical analyses were performed using One-way ANOVA analysis in SAS 2021 software (SAS Institute Inc, Cary, NC, USA). GraphPad Prism (version 8; GraphPad Software, San Diego, CA, USA) was used to generate graphs. *P* < 0.05 was considered statistically significant and *P* < 0.01 was considered extremely significant.

## Results

### Changes in the Serum Biochemical Indices of Piglets Under Starvation

**Figure 1:**
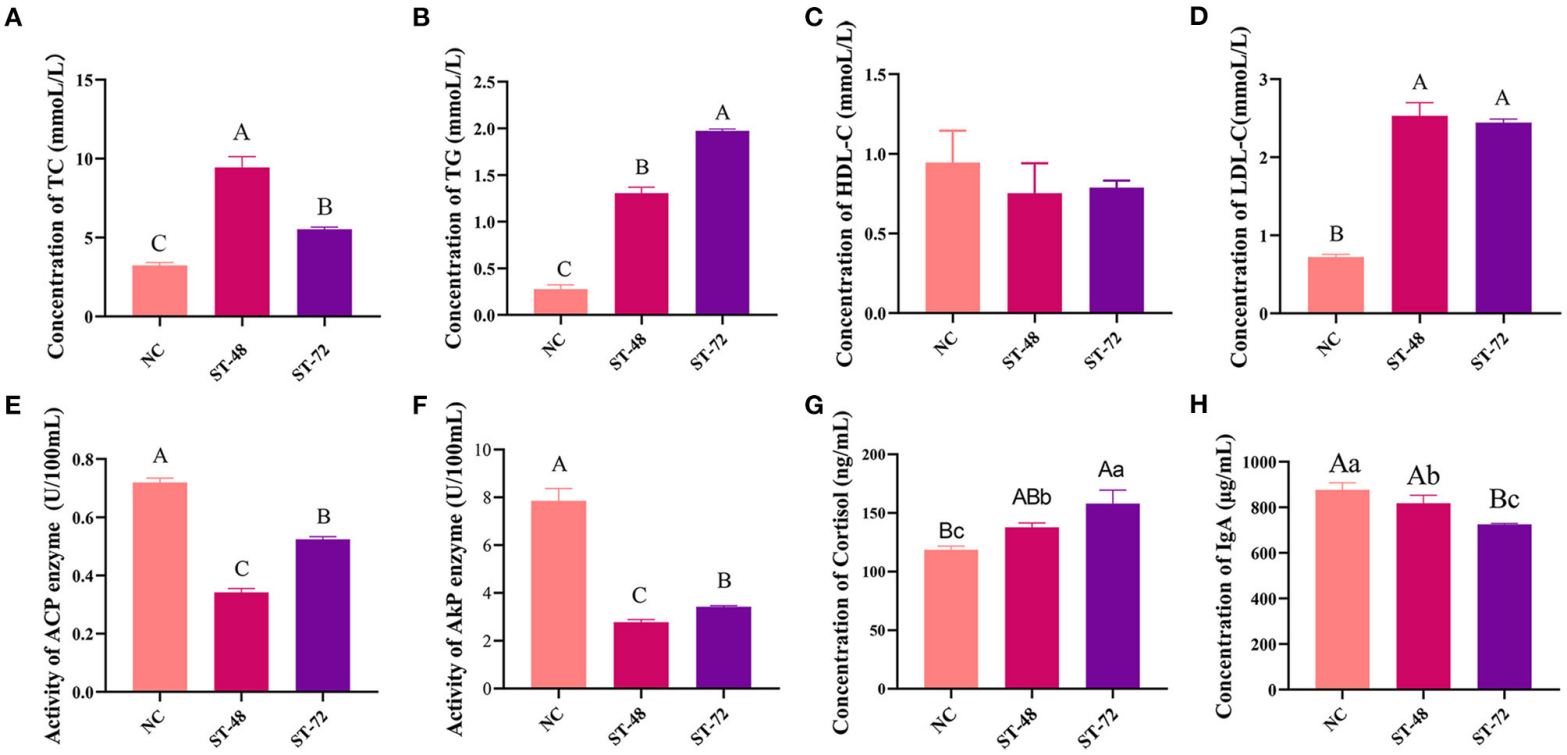
Serum biochemical parameters of **(A)** total cholesterol, **(B)** triglycerides, **(C)** high-density lipoprotein cholesterol, **(D)** low-density lipoprotein cholesterol, **(E)** acid phosphatase, **(F)** alkaline phosphatase, **(G)** cortisol, and **(H)** IgA were measured in the starvation stress groups and normal piglets. Uppercase and lowercase letters indicate significant differences at *P* < 0.01 and *P* < 0.05, respectively, between groups. Data marked with different uppercase letters indicate significant differences (*P* < 0.01), different lowercase letters represent significant differences (*P* < 0.05), respectively.

To evaluate the effect of starvation on piglets, the OD values of TC, TG, HDL-C, LDL-C, ACP, AKP, cortisol, and IgA were measured in the serum (Figure 1). We found that TC and LDL-C were significantly higher after starvation compared with those of NC and were observed to be highest in the ST_48 group (*P* < 0.05). TG and cortisol increased significantly with starvation time and were significantly higher in the ST_72 group than in the other two groups (*P* < 0.01). Contrarily, ACP and AKP enzymes decreased upon starvation and increased with starvation time; however, their concentrations were lower than that of NC (*P* < 0.01). The IgA index decreased gradually with starvation time (*P* < 0.05). HDL-C concentration was not significantly different between the three groups, although cortisol decreased significantly in the ST_48 group (*P* < 0.05). These results suggest that lipid and energy reserves were consumed to maintain the energy needs of the piglet upon starvation.

### Changes in the Intestine Microbiota of Piglets Under Starvation

#### Richness and Diversity of the Intestinal Microbiota

**Figure 2:**
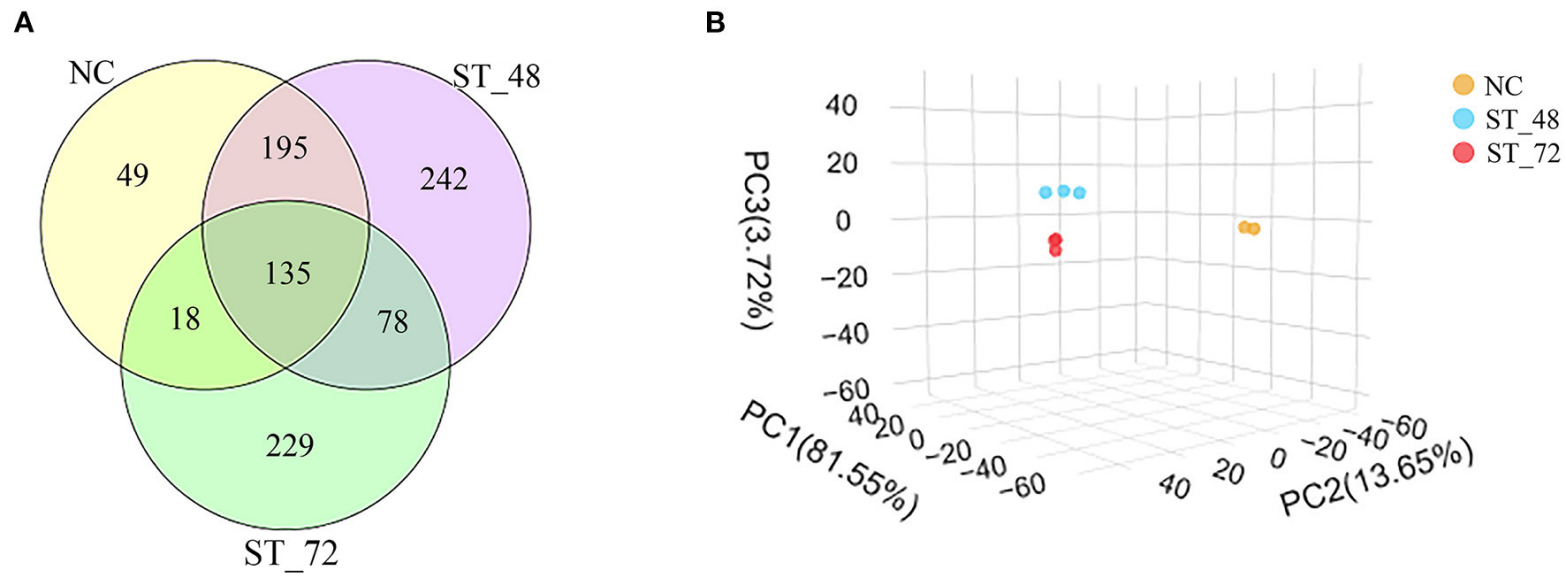
**(A)** Venn diagram showing the number of intestinal genera in the ileum of the NC, ST_48, and ST_72 groups. **(B)** Principal coordinate analysis score plots of OTUs among the NC, ST_48, and ST_72 groups.

**Figure 3:**
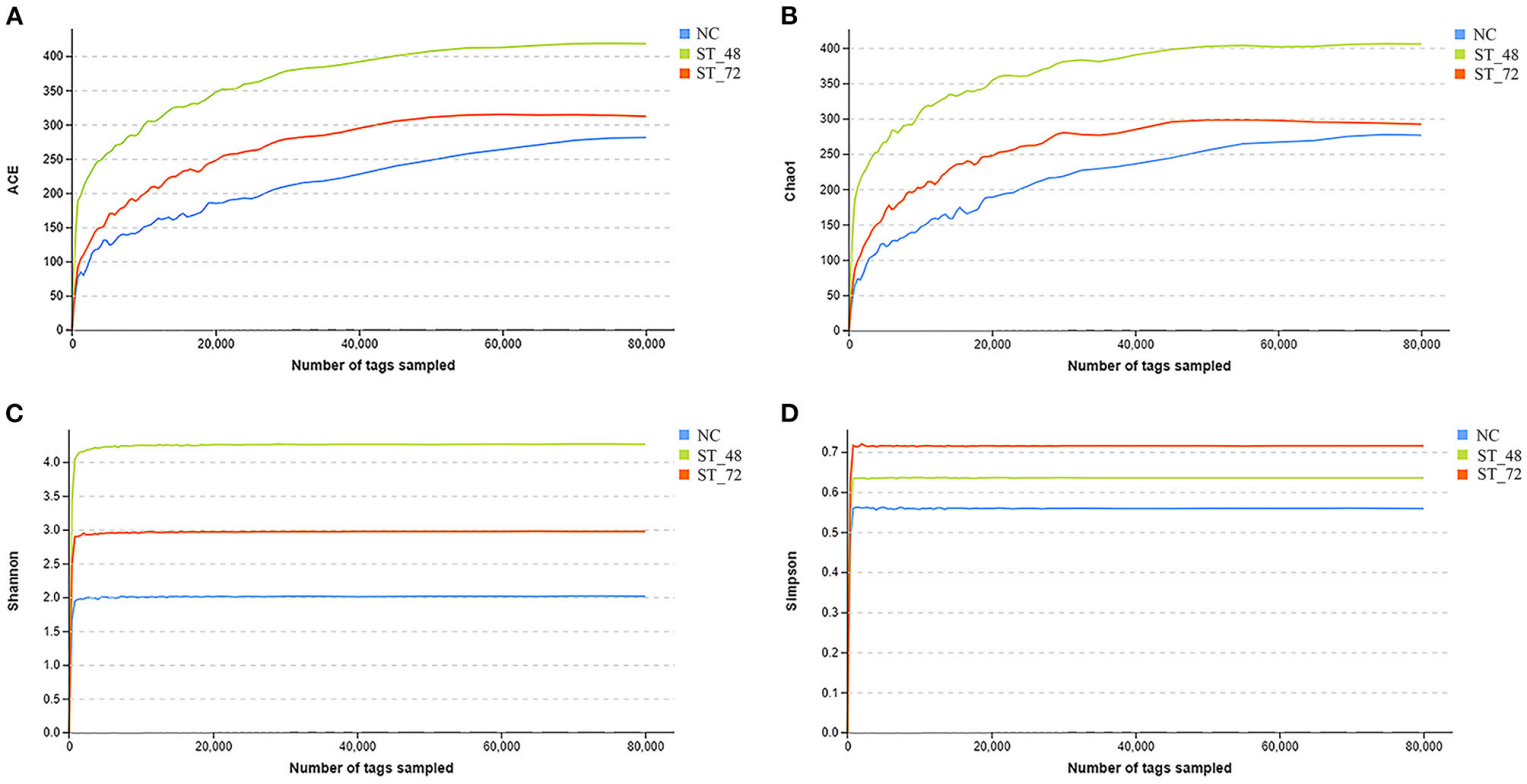
Alpha diversity analysis using **(A)** ACE, **(B)** Chao1, **(C)** Shannon, and **(D)** Simpson indices.

To observe the changes in the intestinal microbiota of piglets under starvation, ileum microbial diversity was analyzed using 16S rRNA sequencing. Over 87% of the samples were sequenced effectively after filtration and *de novo* assembly, proving the reliability of our data (Supplementary Table S1). Because the NC_1 sample was clearly separated from the other two samples of the NC group, NC_1 was not used for subsequent analysis (Supplementary Figure S2). Based on sequence similarity, OTUs were used to classify microbial diversity. We identified 397 OTUs in the NC group, 650 OTUs in the ST_48 group, and 460 OTUs in the ST_72 group (Supplementary Table S2). We found that the number of OTUs specific to the NC groups (12.34%) was lower than that in the ST_48 (37.23%) and ST_72 groups (49.78%). The number of OTUs shared among the three groups was 135 (Figure 2A). The differences between groups were calculated based on PCA. The NC group was separated from the ST_48 and ST_72 groups in the PCA (Figure 2B). Alpha diversity analysis through a rarefaction curve suggested that sequencing data were adequate to cover all microbial communities (Figure 3; Supplementary Table S3). Furthermore, the ACE (Figure 3A), Chao1 (Figure 3B), and Shannon (Figure 3C) indices were highest in the ST_48 group, whereas the Simpson index (Figure 3D) continuously increased and reached its highest value in the ST_72 group. Species diversity and richness increased in the ST_48 group. The changes in the four indices suggested the piglets had more microbiota taxa after starvation for 48 h, and significant differences were observed after starvation for 72 h.

#### Composition of the Intestinal Microbiota

**Figure 4:**
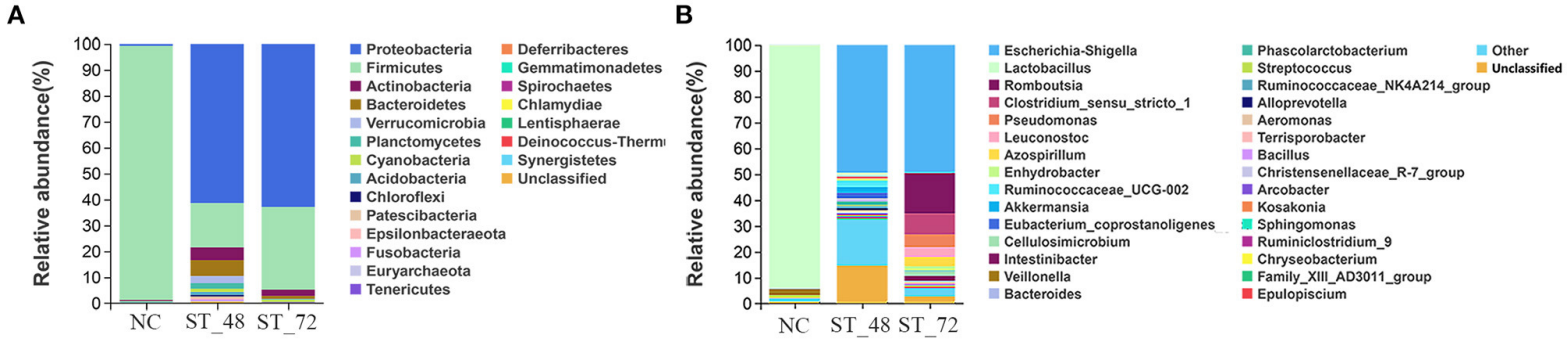
Diversity of intestinal microbiota composition of piglets starved for 48 and 72 h and the control group. Changes in microbial composition of each sample constructed at the **(A)** phylum and **(B)** genus levels (Top 30 in abundance).

Species composition analysis of the microbial community showed that 21 bacteria were identified at the phylum level (Figure 4A; Supplementary Table S4). The dominant phylum in NC was Firmicutes (97.98%), whereas that in the ST_48 and ST_72 groups was Proteobacteria (61.30 and 62.87%, respectively). The abundance of Bacteroidetes decreased in the NC and ST_72 groups (0.15 and 1.14%, respectively) and significantly increased in the ST_48 group (6.29%, *P* < 0.05). One hundred and seventy-one bacteria were identified at the family level, and the top 30 were selected for mapping (Supplementary Figure S2, Supplementary Table S5). *Lactobacteriaceae* was the major family during the weaning process of piglets, accounting for 94.09% of the total abundance in the NC group. We found that the abundance of Enterobacteriaceae increased significantly in the ST_48 (51.01%) and ST_72 groups (49.71%) compared with that in the NC group (0.14%, *P* < 0.05). The abundance of Ruminococcaceae was significantly higher in the ST_48 group (9.25%) compared with that in the NC (0.04%) and ST_72 groups (0.10%, *P* < 0.05). The abundance of *Peptostreptococcaceae* increased significantly in the ST_72 group (18.06%) than that in the NC (0.16%) and ST_48 groups (0.08%, *P* < 0.05). In addition, a total of 311 genera were identified in the three groups (Supplementary Table S6). 142, 233, and 202 genera were annotated in NC, ST_48 and ST_72 groups. The results showed that NC group had the least number of genera in the three groups, the number and abundance of genera in the ST_48 and ST_72 groups changed significantly compared with those in NC. Of these, 116, 72, and 105 genera were presented in NC vs. ST_48 (77 upregulated and 39 downreg-ulated), NC vs. ST_72 (31 upregulated and 41 downregulated), and ST_48 vs. ST_72 (30 upregulated and 75 downregulated), respectively (Supplementary Figure S3). Additionally, the top 30 genera were selected for mapping (Figure 4B). *Lactobacillus* abundance was highest in the NC group (94.09%). A similar *Escherichia-Shigella* abundance was observed in the ST_48 and ST_72 groups (49.45 and 49.53%, respectively), significantly higher than that in the NC group (0.11%, *P* < 0.05). Moreover, some genera were highly abundant in the ST_48 group, including *Ruminococcaceae*_UCG-002 (2.44%) and *Akkermansia* (2.26%). *Romboutsia, Clostridium_sensu_stricto*_*1*, and *Pseudomonas* were highly abundant in the ST_72 group (15.60, 8.08, and 4.88%, respectively).

#### Specific Intestinal Microbiota in Each Group

**Figure 5:**
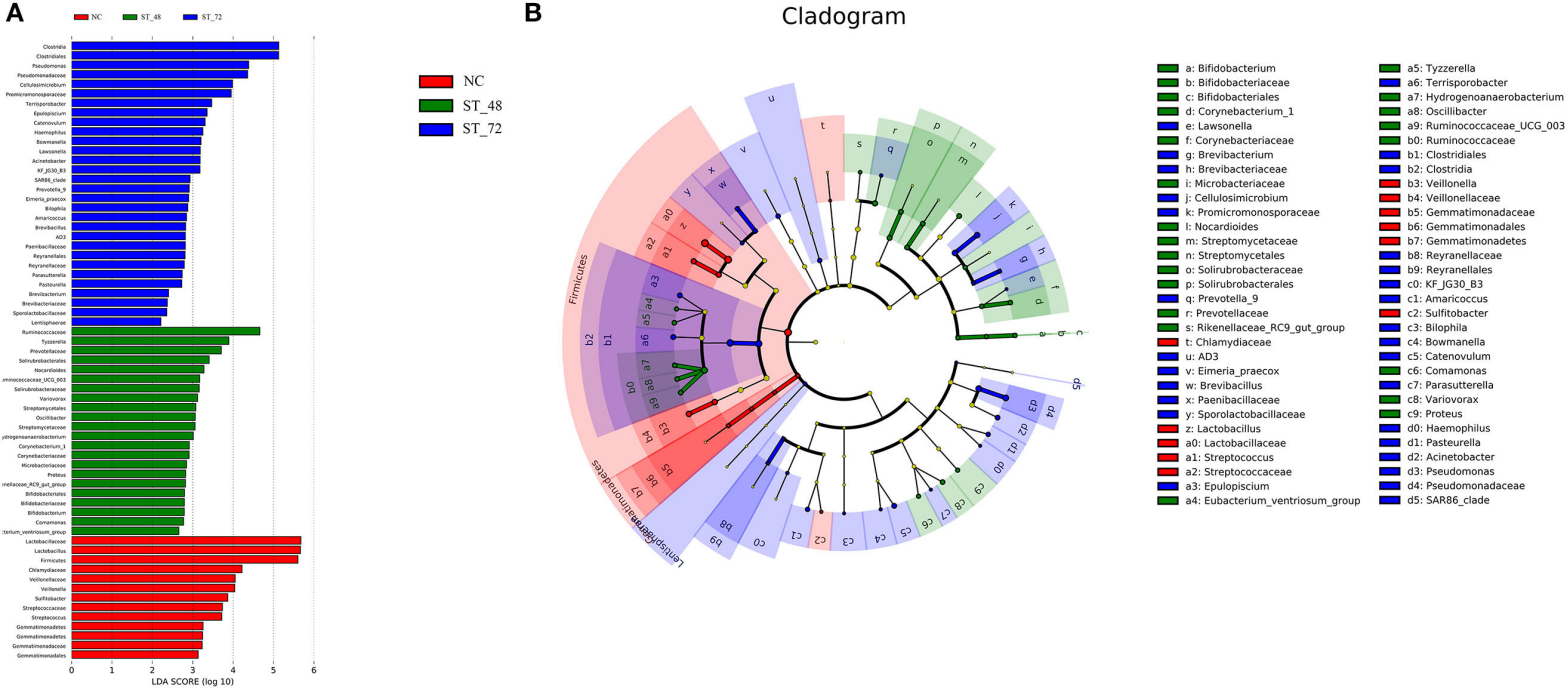
Linear discriminant analysis effect size (LEFse) analysis of intestinal microbiota in piglets. LEFse identified the abundant taxa difference between the NC, ST_48, and ST_72 groups. At the phylum, family, and genus levels, the taxa enriched in NC are presented by a positive LDA score (red), those enriched in the ST_48 group are represented by a positive LDA score (green), and those enriched in the ST_72 group are represented by a positive LDA score (blue). Taxa with *P* < 0.05 and LDA >2 are displayed. **(A)** LDA discriminant results map. **(B)** LEFse multilevel species hierarchical treemap.

To identify specific microbiota after starvation stress, we compared the composition of the intestinal microbiota of the NC, ST_48, and ST_72 groups using LEFse analysis. LEFse analysis revealed 53 discriminative features (LDA > 2, *P* < 0.05; Figure 5; Supplementary Table S7). At the genus level, marked differences among the 32 genera were observed (*P* < 0.05). Specific genera were observed in the NC group, including *Lactobacillus, Veillonella, Streptococcus*, and *Bavariicoccus*. Eighteen specific genera (e.g., *Tyzzerella, Ruminococcaceae*_*UCG*_*003, Variovoram, Corynebacterium_1, Proteus*, and *Rikenellaceae_RC9*) were significantly enriched in the ST_48 group (*P* < 0.05). Meanwhile, 18 specific genera were found in the ST_72 group, including *Pseudomonas, Trisporum, Haemophilus, Acinetobacter, Prevotella_9*, and *Pasteurella* (*P* < 0.05, Figure 5). Most of the specific genera belong to Firmicutes in healthy piglets, and with the extension of starvation time, the specific genera gradually changed to Bacteroidetes and Proteobacteria.

### Relationship Between the Intestinal Microbiota and Biochemical Indices in Piglets

**Figure 6:**
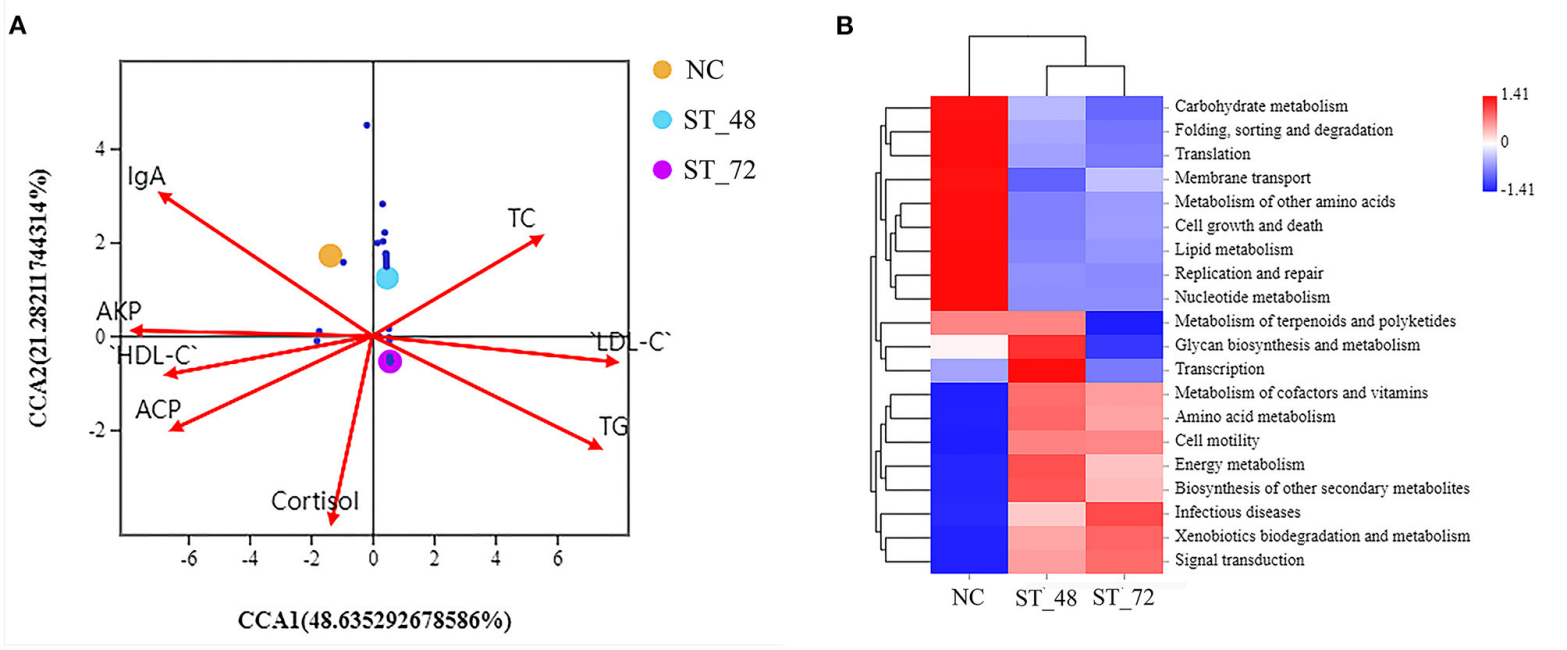
Correlation analysis between different bacteria and environmental factors and pathway enrichment analysis. **(A)** Corresponding sequence diagram of the top 30 genera with significant differences and environmental factors based on CCA analysis. **(B)** KEGG pathway enrichment heatmap showing the alteration in biological processes and metabolisms under starvation stress piglets. Red, positive correlations; blue, negative correlations.

To explore the correlation between the intestinal microbiota and biochemical indices, the CCA method was used to analyze the correlation between the top 30 genera with significant differences (A total of 197 different bacteria were found in the three groups, Supplementary Table S8) and blood indices, such as TC, TG, HDL-C, and LDL-C (Figure 6A; Supplementary Tables S9, S10). These data show that most genera were not separated and concentrated in the central area. Furthermore, CCA analysis showed that *Pseudomonas, Lactobacillus, Akkermansia, Veillonella, Streptococcus, Bacteroides, Escherichia-Shigella, Alloprevotella*, and other genera were significantly related to eight environmental factors (*P* < 0.05). The results indicated that AKP was significantly positively correlated with *Veillonella* and *Lactobacillu*s. TC showed a significant positive correlation with *Alloprevotella*. Moreover, LDL-C and TG levels were significantly positively correlated with *Leuconostoc, Enhydrobacter*, and *Pseudomonas*. Furthermore, the blood indicators HDL-C and cortisol were positively or negatively correlated with different genera.

### Changes in the Function of the Intestinal Microbiota in Piglets Under Starvation Stress

Based on species annotation and OTU abundance, the KEGG pathway was used to annotate the function at level 2, and the abundance of each pathway is displayed in the heatmap (Figure 6B). We observed that the genera were significantly enriched in carbohydrate metabolism, membrane transport, cell growth and death, lipid metabolism, and nucleotide metabolism in the NC group. However, they were significantly increased in cofactor and vitamin metabolism, amino acid metabolism, cellular motility, energy metabolism, biosynthesis of other secondary metabolites, and infectious diseases in the ST_72 and ST_48 groups compared with the control group. Furthermore, compared with the other two groups, metabolism of terpenoids and polyketides and glycan biosynthesis were highly abundant in the ST_48 group.

### Changes in Metabolites in Piglets Under Starvation Stress

**Figure 7:**
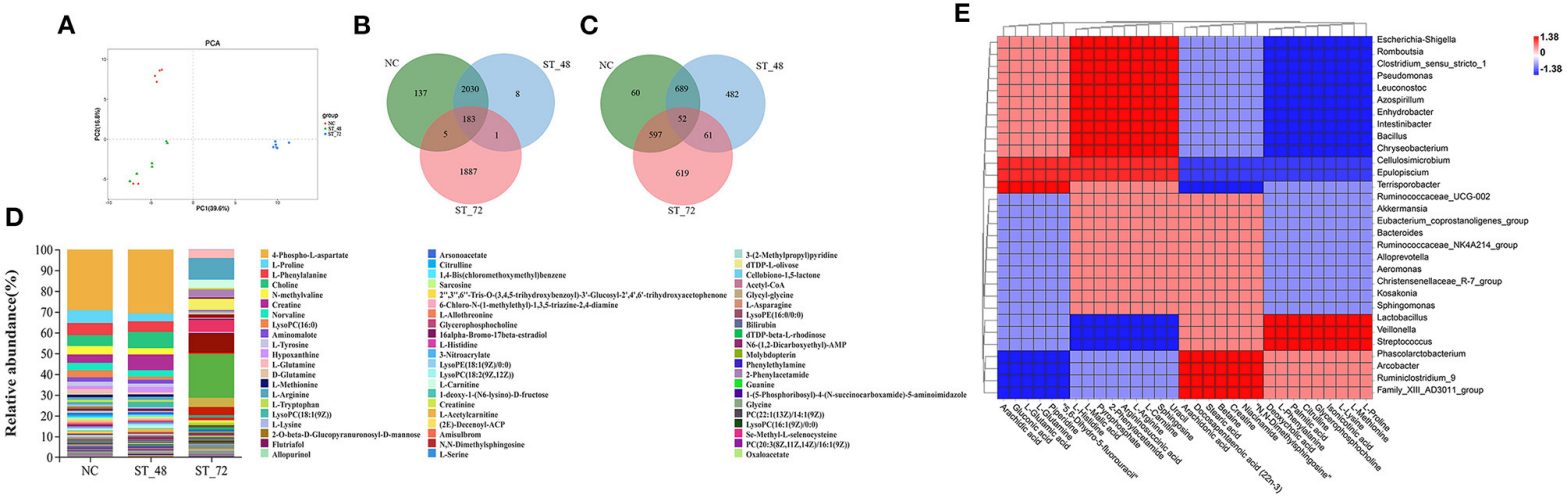
Comparative metabolomics analysis of changes in the intestine metabolites in the starvation and control groups. **(A)** Variation of intestinal metabolite structure represented by PCA of multivariate statistical analysis (MVA). **(B)** Venn diagram showing the metabolite profile between starvation and control groups in positive ion mode (PIM) and **(C)** negative ion mode (NIM). **(D)** Kruskal–wallis test was used to screen the different metabolites in the three groups (The names of the annotated bacteria displayed the top 63). **(E)** The correlation among 30 genera and 30 metabolites is shown using a heatmap. Color intensity represents the magnitude of the correlation. Red, positive correlations; blue, negative correlations.

Simultaneously, we performed a non-targeted metabolomic analysis of the ileal contents in the NC, ST_48, and ST_72 groups. The original data included 5 quality control samples and 18 experimental samples. A total of 6,872 peaks were collected, of which 4,252 were collected in positive ion mode (PIM, Supplementary Table S11) and 2,620 in negative ion mode (NIM, Supplementary Table S12). The MVA of the PCA model was used to observe the classification of different groups in the score chart. The ST_72 group differed significantly from the NC and ST_48 groups. However, two samples in the NC group showed dissociation, which may be due to the individual differences between piglets, so it was not used for subsequent analysis (Figure 7A). Furthermore, the metabolite profiles showed that the piglet metabolites changed significantly after 72 h of starvation. The results of PIM showed there were 183 identical metabolites between the three groups. There were 137 and 8 specific metabolites in the NC and ST_48 groups, respectively, and 2,213 common metabolites between the two groups. However, there were 1,887 specific metabolites in the ST_72 group (Figure 7B). In the NIM results, there was no significant difference in the number of metabolites among the three groups. However, there were only 52 identical metabolites among the three groups, and the specific metabolites among the ST_48 and ST_72 groups were 482 and 619, respectively, which were significantly higher than those in NC (Figure 7C).

### Correlation Analysis Between Different Genera and Metabolites

In this study, differential metabolites were screened based on the VIP values in the OPLS-DA model. Metabolites with VIP > 1 were selected for trend analysis (Supplementary Figure S4, Supplementary Table S13). A total of 238 differential metabolites were found in PIM and NIM, and it was found that the changes in metabolites mainly occurred in the ST_72 group, while there was little difference in the NC and ST_48 groups (Figure 7D). We selected metabolites that were significantly differentially expressed in the three groups and ranked in the top 30, mainly including fatty acids (arachidic, palmitic, and stearic acids), bile acid (deoxycholic acid), some amino acids (L-glutamine, l-phenylalanine, etc.), and derivatives. To further study whether the changes in intestinal metabolites were related to different genera, we used Spearman's correlation analysis to determine the correlation between 30 genera and 30 metabolites and generated a heatmap to clarify this observation (Figure 7E). *Lactobacillus* and *Veillonella* were significantly enriched in the NC group, which were positively correlated with some metabolites (e.g., l-phenylalanine, deoxycholic acid, palmitic acid, *P* < 0.01). *Ruminococcaceae_UCG-002* and *Akkermansia* were enriched in the ST_48 group and positively correlated with creatine, sphingosine, l-glutamine, niacinamide, and arachidonic acid (*P* < 0.01). *Escherichia-Shigella, Romboutsia, Clostridium_sensu_stricto_1*, and *Pseudomonas* had the highest abundance in the ST_72 group and were significantly positively correlated with some metabolites (e.g., sphingosine, l-arginine, gluconic acid, pyrophosphate, l-carnitine; *P* < 0.01). Interestingly, the relationship was opposite between differential genera and metabolites enriched in the NC and ST_72 groups.

### Metabolic Pathway Analysis of the Key Differential Metabolites

**Figure 8:**
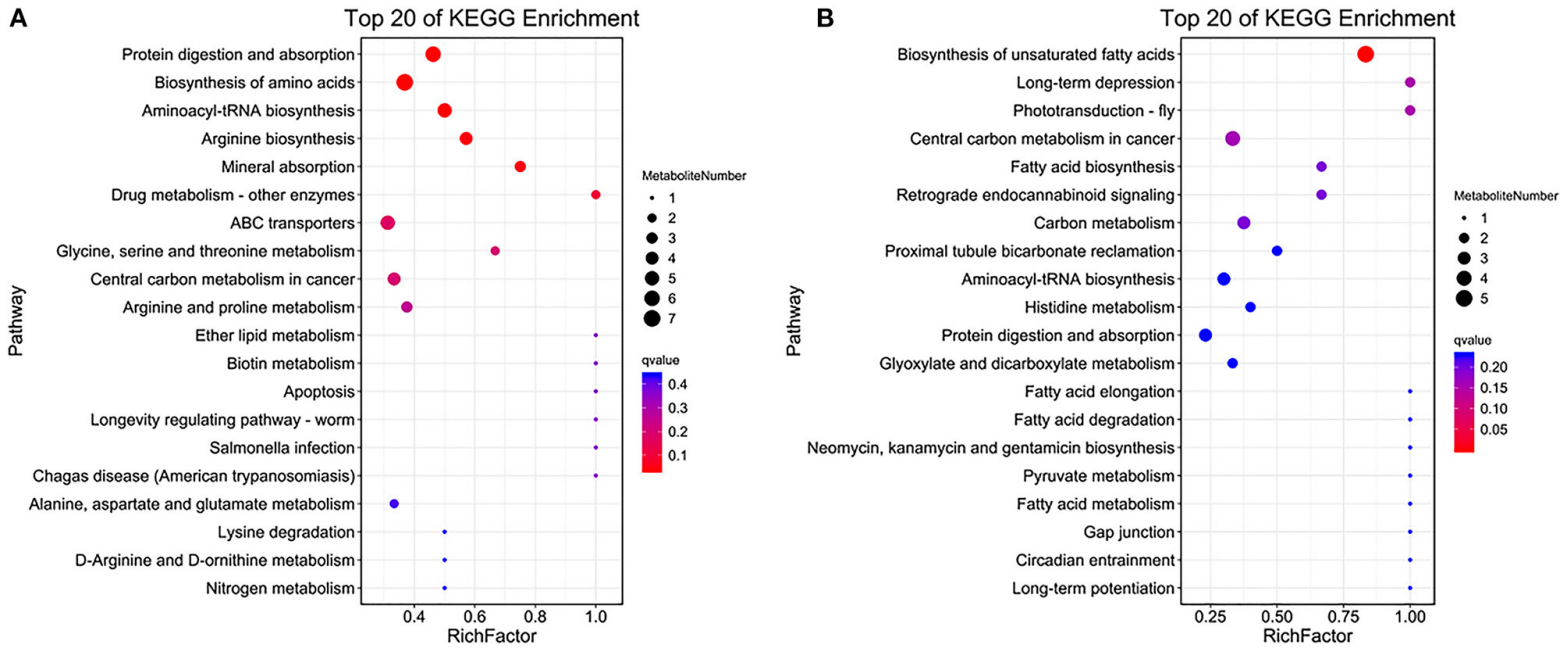
Functional analysis of intestinal metabolites in piglets after starvation. **(A)** PIM and **(B)** NIM.

KEGG pathway analysis of significantly different metabolites was used to clarify the correlation between changes in ileum metabolites and metabolic disorders in weaned piglets after starvation (Figure 8). In the PIM results, the mainly pathways were protein digestion and absorption, metabolism (drug metabolism-other enzymes, ether lipid metabolism and biotin metabolism) in the NC group. Most metabolites were enriched in amino acid metabolism (alanine, aspartate, glutamate metabolism), and longevity regulatory pathway in the ST_48 group. In the ST_72 group, metabolites related to cancer (central carbon metabolism in cancer), disease (Salmonella infection), apoptosis, and amino acid (lysine and arginine) pathways were enriched (Figure 8A). In NIM, the metabolites were significantly enriched in related to fatty acid pathways (fatty acid metabolism, biosynthesis, degradation, etc.) in NC compared with the starvation treatment group. In the ST_48 group, the metabolites were enriched in the lipid metabolism (biosynthesis of unsaturated fatty acids) and immune system (inflammatory mediator regulation of the TRP pathway), etc. Furthermore, the metabolites of the ST_72 group were enriched in cancer (central carbon metabolism in cancer), organic acids metabolism (pyruvate, histidine, glyoxylate and dicarboxylate metabolism), etc. (Figure 8B).

## Discussion

Moderate control of obesity is beneficial to human health; short-term fasting not only helps to control obesity but also provides benefits to body health (Kim et al., 2016; Hu et al., 2018; Subramanian et al., 2020; Christopher et al., 2021). In recent years an increasing number of researchers have performed high-throughput sequencing; however, using single omics approaches resulted in difficulty discovering the increasing expectations of systems biology (Peck et al., 2017; Rothschild et al., 2018). Moreover, most integromics have been applied in human medicine, and there are very few studies on animals (Mihaylova et al., 2018). In the present study, 16S rRNA sequencing technology and LC-MS were used to analyze intestinal microbial diversity and metabolites of Large White piglets at different levels of starvation. Our study provides insight into understanding the energy changes in the body after fasting and whether it can maintain normal metabolism.

Serum biochemical indicators are closely related to animal growth and development, innate immunity, and intestinal health (Jiang et al., 2021). Because starvation can cause blood sugar metabolism disorders, higher concentrations of TC and TG can produce more acetyl-CoA, which increases the oxidation of fatty acids to provide energy to the body (Snipes et al., 1986; Schaefer et al., 2019). Without nutrient intake, the liver will not function properly, leading to an increased risk of the disease and increased LDL-C (Gong et al., 2021). Studies have shown that ACP and AKP were positively correlated with macrophage activity (Gong et al., 2021) and immune capacity (Pröbstel et al., 2020). Studies have shown that an increase in serum cortisol can be an indicator of an animal's stress response (Gong et al., 2015). Moreover, IgA is a key regulator of intestinal mucosal immunity (Pröbstel et al., 2020). Here, TC, TG, cortisol, and LDL-C increased significantly after starvation, and AKP, ACP, and IgA levels decreased significantly after starvation in pig serum. The results showed that the stress response of piglets was enhanced, the metabolic level increased significantly, and immunity decreased after starvation.

Studies have shown that Firmicutes are a marker of obesity, and starvation can significantly reduce the number of Firmicutes in the intestinal tract of rats and hybrid groupers (Breton et al., 2019; Liu et al., 2020). We also observed that the abundance of Firmicutes was significantly reduced after starvation. Most studies have demonstrated that this phylum plays a pathogenic role in enteritis and colon cancer (Yang and Jobin, 2014). In the intestinal tract of an inflammation mouse model, the significantly increased abundance of Proteobacteria reflected a decrease in the permeability of intestinal epithelium and dysregulation of the innate immune response leading to host flora disorder (Na-Ri et al., 2015). Recent studies have shown that probiotics can enhance intestinal barrier function and improve inflammation (Zou and Chen, 2020). *Lactobacillus* has previously been identified as an animal growth promoter (Wang et al., 2019). In our results, the abundance of Proteobacteria increased, and *Lactobacillus* decreased significantly after starvation. It was speculated that the intestinal microflora of the piglets was disturbed, and the intestinal epithelial barrier of the piglets was damaged after starvation, causing intestinal inflammation. Studies have shown that Ruminococcaceae could produce a variety of SCFAs, promote the degradation of polysaccharide fibers, and are negatively correlated with some diseases (e.g., hepatic encephalopathy, NAFLD) (Shang et al., 2016). The abundance of *Rikenellaceae_RC9* was significantly reduced in the intestinal tract of pancreatitis mice (Chen et al., 2017) and broiler necrotizing enteritis (Emami et al., 2021) compared with that in healthy groups. In our study, *Ruminococcaceae_UCG-002* and *Rikenellaceae_RC9* increased significantly in the ST_48 group, indicating that some microbiota could maintain intestinal health after starvation. *Escherichia coli-Shigella* can promote the release of pro-inflammatory factors and induce intestinal inflammation (Cattaneo et al., 2017). *Clostridium _sensu_stricto_1* was significantly higher than NC in mice with necrotizing colitis (Fu et al., 2020). *Pseudomonas* has been confirmed to be the pathogen positively related to TG and LDL-C (Distrutti et al., 2016). These pathogens that affected intestinal immunity were significantly increased in the ST_72 group. However, some probiotics, such as *Romboutsia* and *Leuconostoc*, were also observed. *Romboutsia* was highly abundant in healthy intestinal mucosa and can reduce the risk of colorectal cancer (Mangifesta et al., 2018). *Leuconostoc* has been reported to be a member of lactic acid bacteria and promotes polysaccharide synthesis (Zikmanis et al., 2020). There is a symbiotic relationship between the microbiota and the host, and the microbial diversity and abundance change with gradual depletion of nutrients under starvation.

We selected important metabolites with significant differences to describe and explain their relationship with the screened differential genera. Deoxycholic acid is a bile acid that emulsifies dietary fat, ensures normal cholesterol excretion by hepatocytes, and promotes digestion and absorption in the gastrointestinal tract (Matés et al., 2019). Palmitic acid has also been shown to participate in the mTOR signaling pathway (Marafie et al., 2019). L-phenylalanine has been reported to be positively correlated with the reduction of obesity and the incidence of diabetes (Urpi-Sarda et al., 2019). These three metabolites were highest in the NC group, and most were enriched in some amino acid metabolic pathways. This shows that these metabolites play an important role in the normal growth and development of the body. Niacinamide has been reported to enhance antimicrobial peptides in intestinal epithelial cells and neutrophils to avoid infection (Mathapathi et al., 2017) and was enriched in a pathway related to longevity in the ST_48 group. Arachidonic acid not only plays an important role in lipid metabolism but also produces pro-inflammatory and anti-inflammatory derivatives (Sonnweber et al., 2018). The high concentrations of arachidonic acid in the ST_48 group suggested its regulatory role in body health after starvation. Interestingly, in the present study, most of these metabolites were positively correlated with Lactobacillus and Ruminococc*aceae_UCG-002* in the NC and ST_48 groups but negatively correlated with some pathogenic bacteria (*Escherichia coli-Shigella, Clostridium_sensu_stricto_1, Pseudomonas*, etc.). In addition, many metabolites had high concentrations in the ST-72 group, including sphingosine, L-glutamine, L-arginine, arachidic acid, and pyrophosphate. Sphingolipids and their derivatives can cause inflammatory bowel diseases and autoimmune diseases. Sphingosine is a metabolite of sphingolipid that is associated with the apoptosis pathway, and many studies have shown that it can promote apoptosis and activate inflammatory signaling pathways (Hait et al., 2017). L-glutamic acid has been identified as the pathway for central carbon metabolism in cancer. L-glutamine can inhibit tumor cell activity by promoting cell apoptosis and autophagy in a mouse breast cancer model, which provides a drug target to optimize anticancer effects (Arroyo-Crespo et al., 2018). These results indicate that intestinal homeostasis was impaired due to intestinal epithelial cell apoptosis and inflammation in piglets after 72 h of starvation. Recently, L-arginine has been shown to inhibit TLR4/NF-κB and MAPK signaling pathways and reduce intestinal inflammation induced by endotoxin in pig intestinal epithelial cell lines and mice (Lan et al., 2020). L-arginine has also been shown to enhance the function of intestinal stem cells by increasing the expression of Wnt2b in the primary cells of mice (Hou et al., 2020). Besides the above metabolites, arachidic acid, an unsaturated fatty acid, and pyrophosphate are enriched in the oxidative phosphorylation pathway, which could provide energy for starved piglets. Furthermore, the relationship between most of the differential metabolites of the ST_72 group and the differential genera was opposite to that of the other two groups. Studies have reported that intestinal microbiota and metabolites can affect host metabolism (Santosh et al., 2018), and the metabolites result from the interaction between the microbiota and host (Kimberly et al., 2021). Our study found no significant changes in the species and concentration of intestinal metabolites in piglets starved for 48 h, but there were significant changes in the number and abundance of microbiota at this time. This suggests that when no external nutrients are provided to piglets in the short term, the body can stabilize metabolites to some extent through changes in intestinal microbiota to maintain normal operation and alleviate the effects of short-term fasting (48 h). However, with increasing fasting time (72 h), metabolic changes cannot be stabilized by microbial changes. The increase in the abundance of harmful microbiota (*Escherichia coli-Shigella, Clostridium_sensu_stricto_1, Pseudomonas*, etc.) also promoted sharp increases in the concentration of metabolites such as sphingosine, l-glutamine, and l-arginine, leading to metabolic disorders of the body. The results of this study also explained the “golden 72 hours” rescue theory (Yang et al., 2013) after severe disasters from the perspective of intestinal microecology. In this study, due to consideration of animal welfare, piglets were not starved for a longer period, and the response mechanism of intestinal microecology of piglets under extreme conditions could not be explored, which needs further study.

## Conclusions

In conclusion, 16S rRNA sequencing and LC-MS were used to analyze the changes in intestinal microbial diversity and its metabolites in piglets under starvation stress. The microbial shift from Firmicutes to Bacteroidetes and Proteobacteria was induced by hunger stress. Starvation for 48 h can cause significant changes in intestinal microbial diversity, whereas the concentration of microbial metabolites showed slight changes. When starvation lasted for 72 h, intestinal microbiota and metabolites changed significantly. During short-term or intermittent starvation, piglets may regulate the normal metabolites required by the body by changing the structure of the intestinal microbiota, thus reducing the risk of certain diseases after starvation. After long-term starvation, the intestinal microecology was disturbed. The beneficial bacteria in the intestinal tract were significantly reduced, harmful bacteria were increased, and metabolites related to cytopathic changes and apoptosis were produced, which destroyed the intestinal epithelial barrier and affected the intestinal health of piglets. Intestinal microbiota and their related metabolites may become potential biomarkers for developing disease treatment programs based on the microbiota.

## Data Availability Statement

The datasets presented in this study can be found in online repositories. The names of the repository/repositories and accession number(s) can be found in the article/Supplementary Material.

## Ethics Statement

The animal study was reviewed and approved by the Ethics Committee of the Shanxi Agricultural University. Written informed consent was obtained from the owners for the participation of their animals in this study.

## Author Contributions

YM and PG: designed the study. YM: write manuscripts. PG: wrote, reviewed, and edited. CL, BJ, and JQ: visualized. CC and YY: collected the samples. YZ and GL: organized data. XG, GC, and BL: drew the figures and table. All authors have read and agreed to the published version of the manuscript. All authors contributed to the article and approved the submitted version.

## Funding

This work was funded by the Fund for Shanxi 1331 Project (Grant No. 2017), the National Natural Science Foundation of China (Grant No. 31872336), Project of the Shanxi Province (2021L158), and Basic Research Project of Shanxi Province (Grant No. 20210302124639).

## Conflict of Interest

The authors declare that the research was conducted in the absence of any commercial or financial relationships that could be construed as a potential conflict of interest.

## Publisher's Note

All claims expressed in this article are solely those of the authors and do not necessarily represent those of their affiliated organizations, or those of the publisher, the editors and the reviewers. Any product that may be evaluated in this article, or claim that may be made by its manufacturer, is not guaranteed or endorsed by the publisher.

## Supplementary Material

Supplementary Figure S1 Standard curve of blood indicators. **(A)** IgA and **(B)** Cortisol.

Supplementary Figure S2 Principal coordinate analysis score plots of OTUs among the NC, ST_48, and ST_72 groups (nine samples).

Supplementary Figure S3 The change and number of genera annotated at the genus level. **(A)** Changes of all genera annotated at the genus level (The names of the annotated bacteria displayed the top 63). **(B)** The number of up-regulated and down-regulated bacteria in the three groups after pairwise comparison.

Supplementary Figure S4 Gene trend diagram.

Supplementary Table S1 Data filtering before and after comparative analysis statistical table.

Supplementary Table S2 16SrRNA sequencing annotated Tags and OTUs statistics.

Supplementary Table S3 Alpha diversity index table.

Supplementary Table S4 The abundance of microbiota at the phylum level.

Supplementary Table S5 The abundance of microbiota at the family level.

Supplementary Table S6 The abundance of microbiota at the genus level.

Supplementary Table S7 The specific microbiota were screened and constructed based on LEFse analysis.

Supplementary Table S8 The trend analysis results in relative abundance at the genus level in three groups.

Supplementary Table S9 Coordinate results of explanatory variables based on CCA analysis.

Supplementary Table S10 Coordinate results of response variables based on the CCA analysis.

Supplementary Table S11 Metabolites detected in the positive-ion mode.

Supplementary Table S12 Metabolites detected in the negative-ion mode.

Supplementary Table S13 The screen differential metabolites based on VIP > 1 in the OPLS-DA model.
