# Supplementary figures and images for: Integrated Omics Analysis Reveals Alterations in the Intestinal Microbiota and Metabolites of Piglets After Starvation
Source: Front Microbiol. 2022 Jun 15;13:881099. doi: 10.3389/fmicb.2022.881099 (PMC9240708; doi:10.3389/fmicb.2022.881099)

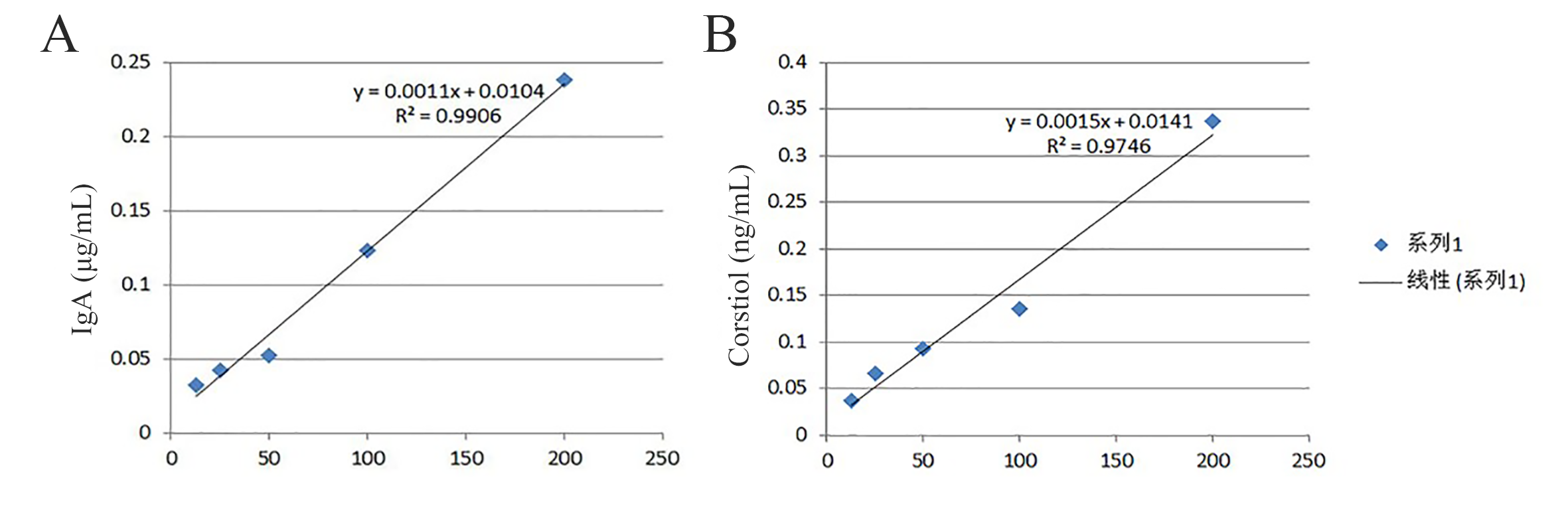

Supplement: Supplementary Figure S1 — Standard curve of blood indicators. (A) IgA and (B) Cortisol. [file Image_1.TIF]

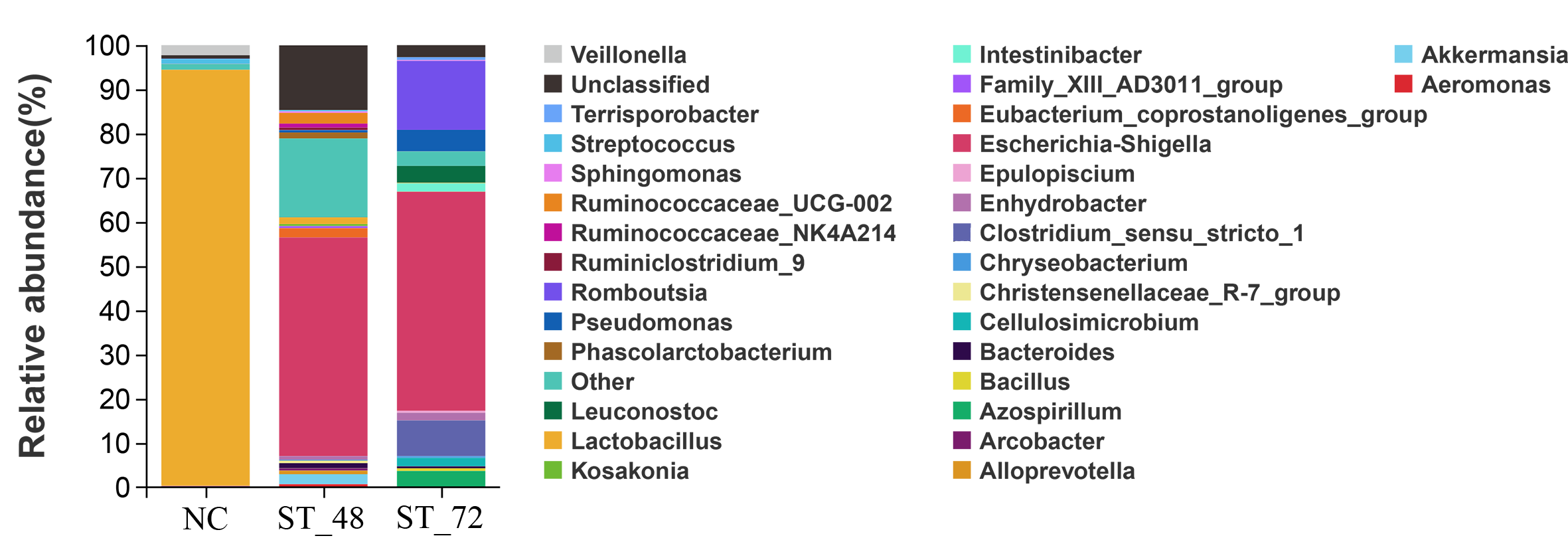

Supplement: Supplementary Figure S2 — Principal coordinate analysis score plots of OTUs among the NC, ST_48, and ST_72 groups (nine samples). [file Image_2.TIF]

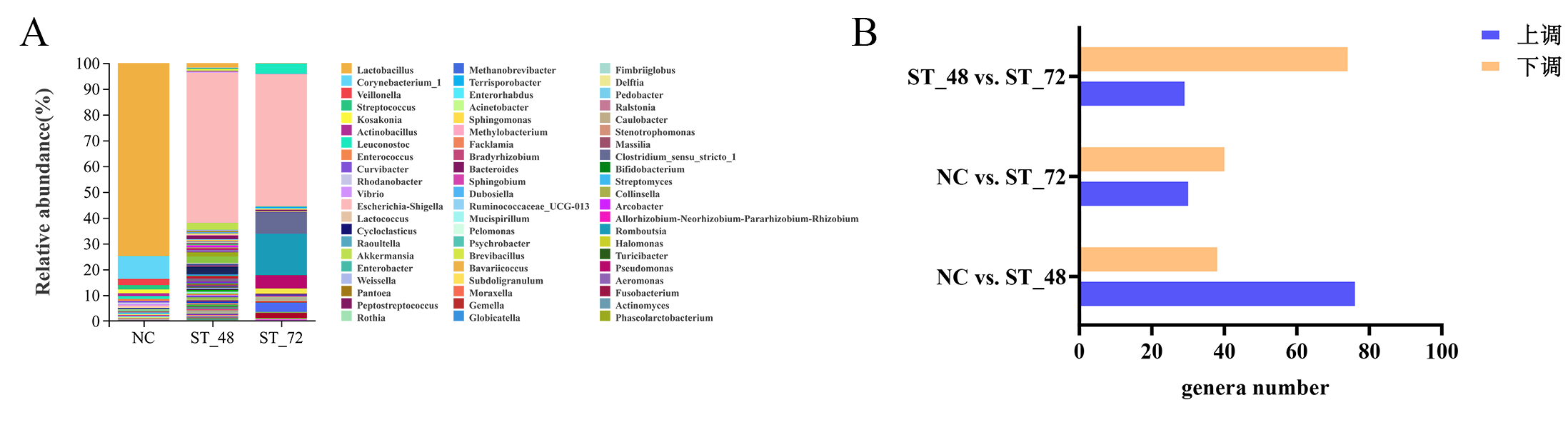

Supplement: Supplementary Figure S3 — The change and number of genera annotated at the genus level. (A) Changes of all genera annotated at the genus level (The names of the annotated bacteria displayed the top 63). (B) The number of up-regulated and down-regulated bacteria in the three groups after pairwise comparison. [file Image_3.TIF]

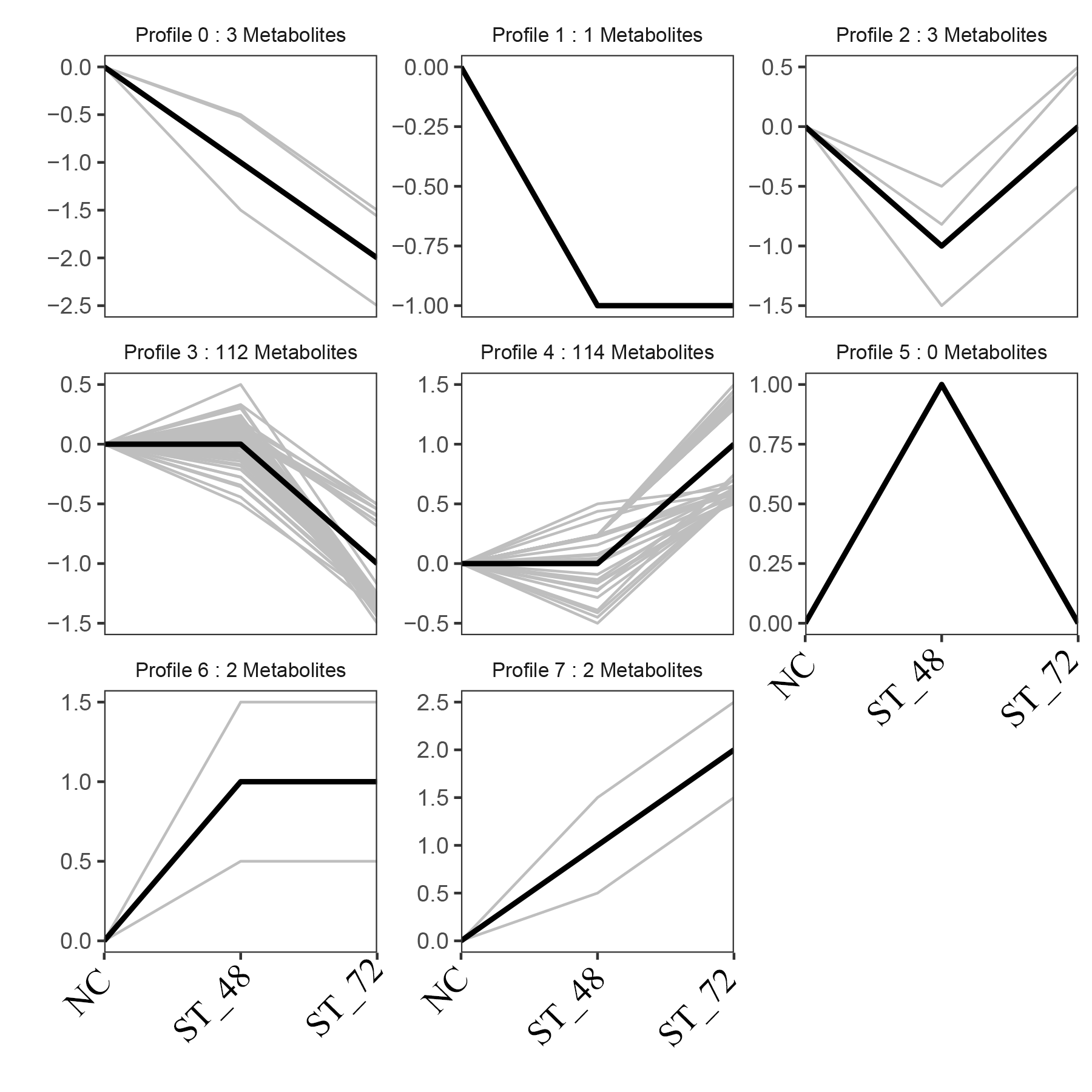

Supplement: Supplementary Figure S4 — Gene trend diagram. [file Image_4.TIF]
